# Correlation of serum subfatin, cthrc1, ctrp3, ctrp6 levels with disease indices in patients with axial spondyloarthritis

**DOI:** 10.1186/s41927-023-00356-5

**Published:** 2023-09-14

**Authors:** İ.Merve B. Uçar, Gökhan Sargin, Ayça Tuzcu, Songül Çildağ, Taşkın Şentürk

**Affiliations:** 1 Department of Internal Medicine, Usak Research and Training Hospital, Rheumatology, Usak, Turkey; 2 Department of Internal Medicine, Aydin Adnan Menderes University, Rheumatology, Aydin, Turkey; 3 Faculty of Medicine, Department of Biochemistry, Aydin Adnan Menderes University, Aydin, Turkey

**Keywords:** Spondyloarthritis, Disease indices, Subfatin, Metrnl, TNF-α, CTRP3, CTHRC1, CTRP6, IL-6, IL-17

## Abstract

**Background:**

The study aimed to investigate novel biomarkers from the C1q TNF superfamily and evaluate their role in autoimmune inflammatory rheumatic diseases with the goal of identifying an effective biomarker to measure clinical disease activity and assess treatment efficacy.

**Methods:**

Sixty-one Axial spondyloarthritis (AxSpa) patients and 30 healthy controls were enrolled in the study. The serum biomarkers subfatin, CTHRC1, CTRP3, CTRP6, IL-6, IL-17, and TNF-α and the disease indices BASDAI, BASFI, MASES, and ASDAS–ESR/CRP were evaluated and compared. The patients were then classified, and their serum biomarkers were assessed according to their ASDAS scores and their treatment regimens.

**Results:**

Among the studied biomarkers, none showed a significant difference between the patients and the healthy controls. Although the difference was not statistically significant, the median values of serum subfatin, CTHRC1, CTRP3, CTRP6, IL-6, IL-17, and TNF-α were all found to be lower in the AxSpa patients than in the healthy controls. Furthermore, once the patients were classified regarding their disease activity, no correlation between the study biomarkers and levels of clinical disease indices was observed. Finally, biological treatments were found to affect the serum concentration of these biomarkers regardless of the level of disease activity.

**Conclusion:**

Novel adipokines and known modulators of inflammation, circulating subfatin, CTHRC1, CTRP3, CTRP6, IL-6, IL-17, and TNF-α levels may play a role in assessing treatment efficacy, especially in those treated with TNF-inhibitors. However, we failed to demonstrate a correlation between clinical disease activity and serum biomarker levels.

## Background

Axial spondyloarthritis (AxSpa) is a chronic inflammatory rheumatic disease leading to chronic back pain. It is important to evaluate clinical findings and monitor disease activity for early diagnosis and effective treatment [1]. The Bath Ankylosing Spondylitis Disease Activity Index (BASDAI), the Bath Ankylosing Spondylitis Functional Index (BASFI), the Bath Ankylosing Spondylitis Metrology Index (BASMI), the Ankylosing Spondylitis Disease Activity Score (ASDAS), the Maastricht Ankylosing Spondylitis Enthesis score (MASES), the erythrocyte sedimentation rate (ESR), and C-reactive protein (CRP) levels are used to evaluate the disease activity and morbidity of ankylosing spondylitis (AS) [2, 3]. BASDAI and BASFI consist of questions evaluating disease symptoms. The MASES score as well as spinal mobility indexes such as BASMI are used in the context of physical examinations, and ASDAS includes ESR and/or CRP [2, 3]. Although ESR and CRP are used to assess disease activity in AxSpa, these parameters can be elevated in many other inflammatory conditions, such as infection and malignancy.

Subfatin (metrnl- or meteorin-like protein) is a novel adipokine that has been shown to play a role in a wide range of physiological functions, including insulin resistance, inflammation, fat thermogenesis, and lipid metabolism. These varying effects are mediated by different intracellular signaling pathways [4]. Subfatin expression has also been shown to increase in hypertrophic chondrocytes, osteoblasts, and synovium in inflammatory arthritis [5]. Recent research highlighted the relationship between subfatin and enthesis inflammation in psoriatic arthritis patients, with a synergistic increase of subfatin levels with serum IL-6, IL-17, and TNF-α levels [5]. Collagen triple helix repeat-containing protein-1 (CTHRC1) is a soluble protein expressed by osteoclasts targeting stromal cells to induce osteoblast differentiation, which results in new bone formation [6]. Murine and human studies have shown increased CTHRC1 levels in inflammatory arthritis and a correlation with inflammatory cytokines, thus suggesting its use as a diagnostic tool [7]. C1q/TNF-related protein-3 (CTRP3) is also named cartducin due to its functions in cartilage growth; it also has an inhibitory role in osteoclast differentiation. It has been suggested that CTRP3 may play a key role in the healing and remodeling of both bone and cartilage in arthritis [8]. C1q/TNF-related protein-6 (CTRP6) is another member of the CTRP family with similar features in glucose and lipid metabolism regulation and anti-inflammatory response. A murine study suggested that CTRP6 may be used both to monitor disease activity and to treat rheumatoid arthritis (RA) [9]. In addition, IL-6, IL-17, and TNF-α play a role in the determination of disease activity [10]. IL-6 was found to correlate with laboratory markers such as ESR and CRP, whereas IL-17 was more likely to be correlated with disease activity [11, 12]. Apart from a few clinical studies on RA patients and murine models of RA, we could not find any previous studies focusing on AS patients and the serum levels of these biomarkers.

In this study, we aimed to evaluate serum subfatin, CTHRC1, CTRP3, CTRP6, IL-6, IL-17, and TNF-α in an AxSpa cohort and correlate the levels with disease activity.

## Methods

A total of 61 AxSpa patients and 30 healthy individuals were included in this cross-sectional study between May-July 2021. AxSpa was diagnosed according to the revised classification criteria of the Assessment of SpondyloArthritis International Society (ASAS) [13]. Patients were excluded if they had infection, malignancy, inflammatory rheumatic diseases other than AxSpa, or were younger than 18 years. Participants provided demographic data, and their ESR, CRP, subfatin, CTHRC1, CTRP3, CTRP6, IL-6, IL-17, and TNF-α were evaluated. Disease activity and morbidity was evaluated with BASDAI, BASFI, BASMI, ASDAS-CRP, and MASES. Healthy controls were also recruited from our rheumatology clinic, whose physical examination, laboratory and/or radiologic work-up did not reveal any pathology.

The participants’ blood samples were collected in heparinized tubes and transferred to the laboratory within one hour of collection. The venous blood samples were then centrifuged at 1,000 x g for 10 min and stored at -80^o^ C until analysis. Commercially available enzyme-linked immunosorbent assay (ELISA) kits for each biomarker (subfatin, CTHRC1, CTRP3, CTRP6, IL-6, IL-17, and TNF-alpha) were used to perform quantification according to the manufacturer’s recommended protocols (BT Lab-Jiaxing Korain Biotech, Zheijang, China). The test results were then reported in units of either ng/mL or pg/mL. Blood sample collection and the disease activity evaluations were conducted on the same visit.

PASW for Windows version 21.0 (SPSS Inc., Chicago, IL, USA) was used for statistical analyses. The data were given as numbers, frequency, mean ± standard deviation, and/or median [25–75p] value. The chi-squared test and Fisher’s exact test were used to analyze categorical data and independence between variables. T-test was conducted to compare cytokine levels between AxSpa and healthy controls. Mann-Whitney U was used in the comparison of different treatment regimens. Kruskal-Wallis H was used in the comparison of cytokine levels in AxSpa patients according to ASDAS disease activity scores. Pearson correlation test was used to establish a correlation with disease indices and serum biomarkers. A p-value below 0.05 was considered statistically significant.

## Results

Demographics of AxSpa patients were as follows: 33 males, 28 females; mean age was 40 ± 10 years. The control group had similar age and gender distribution. All subjects were Caucasian and HLA-B27 positivity was 78.7% in the AxSpa group. Median ESR and CRP of AxSpa patients, though under treatment, were slightly elevated than healthy controls; 18 [8–34] mm/h and 2 [2–9] mg/dL compared to 6 [5–15] mm/h and 2 [2–2]mg/dL respectively. Therapeutic regimens of the patients were classified under three main categories of conventional synthetic disease modifying anti-rheumatic drugs (cDMARD) AS sulfasalazine, TNF-alpha inhibitors [Etanercept (n = 14), Adalimumab (n = 13), Certolizumab (n = 8), Golimumab (n = 4), Infliximab (n = 4)], and non-steroidal anti-inflammatory drugs (NSAID). Demographic parameters of the patients and the control group, along with their treatment and laboratory parameters were shown in Table 1.

**Table 1:** Demographic and laboratory data of AxSpa patients compared with the control group

	AxSpa Patients (n = 61)	Healthy Controls (n = 30)	p-value
Age (years)	40 ± 10	37 ± 11	0.49
Male/Female	33/28	15/15	0.65
BMI (kg/m^2^)	24.9 ± 3	24.2 ± 3	0.53
Treatment			
• cDMARD • TNFi • NSAID	25% 70.5% 98%	N/A N/A N/A	-
ESR (mm/hr)	18 [8–34]	6 [5–15]	**< 0.01**
CRP (mg/L)	2 [2–9]	2 [2–2]	**< 0.01**
HLA-B27 Positivity	78.7%	N/A	-
Subfatin (ng/mL)	0.68 [0.4-1]	0.75 [0.5–1.2]	0.40
CTHRC1 (ng/mL)	55 [40–103]	66 [44–128]	0.35
CTRP3 (ng/mL)	1.9 [1.7–2.2]	2.1 [1.7–2.8]	0.09
CTRP6 (ng/mL)	2 [1.3–2.9]	2.3 [1.3–6.1]	0.47
IL-6 (pg/mL)	71 [55–87]	78 [62–129]	0.15
IL-17 (pg/mL)	21 [16–36]	30 [20–58]	0.19
TNF-α (pg/mL)	98 [83–142]	108 [80–152]	0.97

*Data are given in median [25-75p] or mean ± SD

**ESR: Erythrocyte Sedimentation Rate; CRP: C-reactive protein; CTHRC1: Collagen triple helix repeat-containing protein-1; CTRP3: C1q/TNF-related protein-3; CTRP6: C1q/TNF-related protein-6; TNF: Tumor necrosis factor

Median scores were calculated as 3.3[2.4–5.2] for BASDAI and 2.3[2.0-3.4] for ASDAS-CRP, 1.8 [0.3–4.8] for BASFI, 1 [0–4] for BASMI, and 1 [0–3] for MASES. Patients were then classified into four groups inactive, low, high, and very high disease activity according to ASDAS-CRP cut-off scores, 10% of the subjects were classified as inactive, 25% with low disease activity, 44% with high disease activity, and 21% with very high disease activity.

In AxSpa, median serum levels of subfatin, CTHRC1, CTRP3, CTRP6 were calculated as 0.68 [0.4-1] ng/mL, 55 [40–103] ng/mL, 1.9 [1.7–2.2] ng/mL, 2 [1.3–2.9] ng/mL respectively. Serum cytokine levels of IL-6, IL-17 and TNF-alpha were also found as; 71 [55–87] pg/mL, 21 [16–36] pg/mL, 98 [83–142] pg/mL respectively. Serum levels of the examined biomarkers did not show any significant difference between the study groups. However, a positive correlation was found between the biomarkers(p < 0.001). Furthermore, examined biomarkers failed to establish a distinction between different disease activities. Among the indices, BASDAI was positively correlated with BASFI, ASDAS and MASES scores, BASMI was positively correlated with BASFI and ASDAS scores (p < 0.01). ASDAS-CRP (or ESR) was the only activity score that is positively correlated with other indices. Detailed comparisons of cytokine levels of the study groups according to ASDAS disease activity scores were summarized in Table 2.

**Table 2:** Cytokine levels in AxSpa patients according to ASDAS disease activity scores

	Inactive disease	Low Activity	High Activity	Very High Activity	p-value
Number of subjects	6	15	27	13	-
Age (years)	40 ± 11	39 ± 10	42 ± 10	38 ± 8	0.67
ESR (mm/hr)	10 [2–13]	8 [6–14]	20 [11–31]	41 [31–59]	**< 0.01**
CRP (mg/L)	2 [2–2]	2 [2–2]	2.4 [2-9.1]	36.2 [7.2–43]	**< 0.01**
Subfatin (ng/mL)	0.5 [0.4–1.8]	0.7 [0.4–1.1]	0.6 [0.5–0.9]	0.8 [0.4–1.4]	0.82
CTHRC1 (ng/mL)	55 [40–162]	65 [39–95]	55 [39–84]	51 [47–125]	0.98
CTRP3 (ng/mL)	1.7 [1.6-3]	2 [1.8–2.4]	1.9 [1.7–2.1]	1.9 [1.8-3]	0.81
CTRP6 (ng/mL)	0.9 [0.4–4.5]	1.9 [1.4–2.9]	2.1 [1.4–2.6]	2.22 [1.6–5.9]	0.48
IL-6 (pg/mL)	89 [69–232]	73[51–82]	71 [58–79]	69 [51–135]	0.45
IL-17 (pg/mL)	17 [15–60]	28 [19–45]	19 [15–33]	26 [21–55]	0.35
TNF-alpha (pg/mL)	110 [83–168]	98 [83–142]	96 [80–129]	106 [94–146}	0.96
BASDAI (0–10)	1.8 [1.7–2.8]	2.2 [1.6–3.2]	4.5 [2.8–5.6]	4.3 [2.8–6.4]	**< 0.01**
BASFI (0–10)	0 [0-0.6]	1 [0-3.1]	2.6 [1–5]	3.4 [2.4-7]	**< 0.01**
BASMI (0–10)	1 [0–2]	0 [0–2]	1 [0–4]	4 [0–7]	0.15
MASES (0–13)	1 [0–1]	0 [0–3]	2 [0–5]	0 [0–3]	0.64
ASDAS-CRP	1 [0.7-1]	1.9 [1.6-2]	2.6 [2.3-3]	3.9 [3.7–4.1]	**< 0.01**
ASDAS-ESH	1.2 [0.7–1.2]	1.8 [1.5–2.3]	2.9 [2.4–3.2]	3.8 [3.6–4.1]	**< 0.01**

*Data are given in median [25-75p] or mean ± SD.

**ESR: Erythrocyte Sedimentation Rate; CRP: C-reactive protein; CTHRC1: Collagen triple helix repeat-containing protein-1; CTRP3: C1q/TNF-related protein-3; CTRP6: C1q/TNF-related protein-6; TNF: Tumor necrosis factor; BASDAI: Bath Ankylosing Spondylitis Disease Activity *Index*; BASFI: Bath Ankylosing Spondylitis Functional *Index; BASMI*: Bath Ankylosing Spondylitis Metrology *Index; MASES*: Maastricht Ankylosing Spondylitis Enthesitis Score; ASDAS-CRP: Ankylosing Spondylitis Disease Activity Score-CRP.

When AxSpa patients were categorized according to their treatment regimen, patients who are on TNFi have significantly lower serum subfatin, CTRP3, CTRP6, IL-17 and TNF-α levels, whereas disease indices were similar. The patients who are on biological agents were significantly older than cDMARD group. The comparison of different treatment groups are summarized in Table 3.

**Table 3:** The comparison between different treatment groups

	TNF-inhibitors (n = 43)	cDMARD (n = 18)	p-value
Age (years)	43 ± 9	34 ± 9	**0.02**
ESR (mm/hr)	14 [8–35]	21 [9–34]	0.72
CRP (mg/L)	2 [2–9]	4.4 [2–10]	0.61
Subfatin (ng/mL)	0.57 [0.4–0.8]	0.89 [0.6–1.7]	**0.03**
CTHRC1 (ng/mL)	54 [39–89]	71 [40–181]	0.33
CTRP3 (ng/mL)	1.9 [1.7-2]	2.2 [1.8-3]	**0.03**
CTRP6 (ng/mL)	1.92 [1.1–2.6]	2.85 [1.5–6.8]	**0.03**
IL-6 (pg/mL)	70 [55–80]	73 [55–138]	0.35
IL-17 (pg/mL)	19 [15–32]	33 [21–55]	**0.04**
TNF-α (pg/mL)	93 [76–128]	118 [97–163]	**0.01**
ASDAS-CRP	2.4 ± 1.0	2.8 ± 0.9	0.16
BASDAI	3.4 ± 1.8	4.1 ± 1.7	0.21

*Data are given in median [25-75p] or mean ± SD

**ESR: Erythrocyte sedimentation rate; CRP: C-reactive protein; CTHRC1: Collagen triple helix repeat-containing protein-1; CTRP3: C1q/TNF-related protein-3; CTRP6: C1q/TNF-related protein-6; TNF: Tumor necrosis factor; BASDAI: Bath Ankylosing Spondylitis Disease Activity *Index*; ASDAS-CRP: Ankylosing Spondylitis Disease Activity Score-CRP

## Discussion

In this study, we primarily aimed to present the relationship between some recently highlighted novel biomarkers and AxSpa disease activity, we also aimed to evluate the relation between serum biomarkers and other disease indices. Our results show that serum markers of subfatin, TNF-α, IL-6, IL-17, CTHRC1, CTRP3, and CTRP6 in AxSpa patients who are being treated either by TNFi or cDMARD do not significantly differ from the healthy controls. Contrarily, median values of these biomarkers were found to be lower in treated AxSpa patients than in the control group. Since most of our patients were on biological agents, these lower values are most probably associated with their treatment regimen. However, the lack of any significant difference in biomarker levels between different groups of disease activity implies that serum levels of these biomarkers may also be regulated by additional pathways.

It was previously shown in the literature that interleukin levels do not necessarily correlate with disease activity in various spondyloarthritis types [11–15]. However, some of these studies revealed increased levels of IL-6 and IL-17 in AxSpa patients compared with healthy individuals and a study also showed a positive correlation between IL-17 levels and BASDAI scores in 49 AxSpa patients [15]. Braga et al. also reported in a cohort of 149 AxSpa patients that serum TNF-α and IL-17 levels do not differ from healthy controls, but they underlined the fact that all their subjects were being treated with TNFi [16]. On the contrary, we found that patients who are using biological agents compared with patients who are on cDMARDs have lower serum TNF-α and IL-17 levels in addition to lower subfatin, CTRP3, and CTRP6 levels, despite similar disease activity parameters. It should also be noted that subjects on biological treatments are slightly older than their counterparts and the serum levels of subfatin, CTRP6, and especially CTRP3 have all been reported to be influenced by age and metabolic status as they both are expressed throughout the body [17, 18]. Although many in vitro effects, such as inflammatory signal attenuation and promotion of cellular differentiation, have been demonstrated earlier by many studies, there is a lack of in vivo data, especially in autoimmune and autoinflammatory diseases [9, 17–19].

To the best of our knowledge, this is the first study to evaluate serum levels of subfatin, CTHRC1, CTRP3, and CTRP6 and their relationship with disease indices in AxSpa. Our results support the fact that serum biomarkers could not be relied on when it comes to assessing the disease activity or morbidity, and as each biological agent blocks a specific inflammatory pathway, the use of TNFi makes the evaluation of some biomarkers even more complicated [20]. A major limitation of our study is that none of our patients were treatment naïve, and we did not take disease or treatment duration into consideration. A relatively small sample is also another limitation of our study. Last but not least, we observed that the serum levels of these biomarkers in healthy subjects vary between various studies, this may be attributable to different kit manufacturers [17]. In conclusion, novel biomarkers subfatin, CTHRC1, CTRP3, and CTRP6 may play a future role in the evaluation of the efficacy of biological agents however, studies conducted on larger cohorts involving treatment naïve patients are crucial in this pursuit.

## Data Availability

The datastes of this study are available from the corresponding author, ,I.M.B.U, upon reasonable request.
